# Enhanced Fatty Acid Oxidation and FATP4 Protein Expression after Endurance Exercise Training in Human Skeletal Muscle

**DOI:** 10.1371/journal.pone.0029391

**Published:** 2012-01-03

**Authors:** Jacob Jeppesen, Andreas B. Jordy, Kim A. Sjøberg, Joachim Füllekrug, Andreas Stahl, Lars Nybo, Bente Kiens

**Affiliations:** 1 Molecular Physiology Group, Department of Exercise and Sport Sciences, University of Copenhagen, Copenhagen, Denmark; 2 Integrated Physiology, Department of Exercise and Sport Sciences, University of Copenhagen, Copenhagen, Denmark; 3 Molecular Cell Biology Laboratory, Internal Medicine IV, University of Heidelberg, Heidelberg, Germany; 4 Department of Nutritional Sciences and Toxicology, University of California, Berkeley, California, United States of America; Montreal Diabetes Research Center, Canada

## Abstract

FATP1 and FATP4 appear to be important for the cellular uptake and handling of long chain fatty acids (LCFA). These findings were obtained from loss- or gain of function models. However, reports on FATP1 and FATP4 in human skeletal muscle are limited. Aerobic training enhances lipid oxidation; however, it is not known whether this involves up-regulation of FATP1 and FATP4 protein. Therefore, the aim of this project was to investigate FATP1 and FATP4 protein expression in the vastus lateralis muscle from healthy human individuals and to what extent FATP1 and FATP4 protein expression were affected by an increased fuel demand induced by exercise training. Eight young healthy males were recruited to the study. All subjects were non smokers and did not participate in regular physical activity (<1 time per week for the past 6 months, VO_2peak_ 3.4±0.1 l O_2_ min^−1^). Subjects underwent an 8 week supervised aerobic training program. Training induced an increase in VO_2peak_ from 3.4±0.1 to 3.9±0.1 l min^−1^ and citrate synthase activity was increased from 53.7±2.5 to 80.8±3.7 µmol g^−1^ min^−1^. The protein content of FATP4 was increased by 33%, whereas FATP1 protein content was reduced by 20%. Interestingly, at the end of the training intervention a significant association (r^2^ = 0.74) between the observed increase in skeletal muscle FATP4 protein expression and lipid oxidation during a 120 min endurance exercise test was observed. In conclusion, based on the present findings it is suggested that FATP1 and FATP4 proteins perform different functional roles in handling LCFA in skeletal muscle with FATP4 apparently more important as a lipid transport protein directing lipids for lipid oxidation.

## Introduction

Skeletal muscle expresses several membrane bound lipid binding proteins such as the plasma membrane fatty acid binding protein (FABPpm) [1], fatty acid transport protein (FATP) 1 and 4 [2], [3], [4], [5], fatty acid translocase CD36 (FAT/CD36) [6] and, in addition, two intracellular proteins, the cytosolic fatty acid binding protein (FABPc) [7] and the acyl-CoA binding protein (ACBP) [8], which have been shown to be important in cellular LCFA handling [9], [10], [11]. Furthermore, two small integral membrane proteins, Caveolin 1 and Caveolin 3, critical in the formation of caveolae in endothelia cells (Caveolin 1) [12] and skeletal muscle (Caveolin 3) [13], were recently shown to have an important role in regulation of LCFA metabolism [14], [15]. Most of the lipid binding proteins have been identified in human skeletal muscle on the protein level [16], [17], [18], [19], [20], [21]. However, whether protein, and not only mRNA, levels of FATP1 and FATP4, the major FATP isoforms expressed in rodent skeletal muscle [3], [4], [5], [22], are expressed in human skeletal muscle, have yet to be addressed. The generation of genetic FATP1 and FATP4 loss-of-function models (i.e. FATP1 KO- and FATP4 heterozygote mice) revealed an important role in LCFA uptake in muscle cells [23] and enterocytes [2], respectively. However, the mechanism by which these proteins facilitate LCFA uptake in skeletal muscle cells is unclear. Detailed membrane topology analysis suggests that FATP1 protein has at least one transmembrane and multiple membrane associated domains [24]. FATP4 appears to share this transmembrane domain topology [25], and an overall sequence similarity [26] suggests it is common to all FATP family members [27]. Importantly, FATP1 and FATP4 were shown to possess long chain acyl CoA synthetase activity [28], [29]. Taken together, the findings suggest that FATP1 and FATP4 induced activation of LCFA, by the formation of fatty acyl-CoA once LCFA is taken up by cells or released from the intramyocellular triacylglycerol (IMTG) pool, could be a major contributor to the regulation of LCFA metabolism in skeletal muscle.

Under physiological conditions with increased cellular demand of LCFA for energy turnover, such as exercise training, FABPpm protein expression has consistently been shown to be increased in human skeletal muscle [16], [19], [20], [21], whereas reports of the effect of exercise training on FAT/CD36 protein expression are contradictory [19], [20], [21], [30]. Furthermore, FABPpm and FAT/CD36 protein expression were increased in vastus lateralis muscle from human subjects after 4–7 weeks on an isocaloric high fat diet [31]. This could indicate that LCFA flux in human skeletal muscle is associated with an increased FABPpm and FAT/CD36 protein expression. In contrast, it is unknown how increased LCFA turnover affects FATP1 and/or FATP4 protein expression. Therefore the main purpose of this study was to identify if human skeletal muscle expresses FATP1 and FATP4 at the protein level and furthermore, whether these proteins were affected by an increased fuel demand induced by exercise training. We hypothesized that endurance exercise training, which is known to increase the potential for an enhanced systemic LCFA utilization [32], [33], [34], [35] and skeletal muscle lipolytic capacity [36], [37], [38], [39], will provide adaptations in FATP1 and FATP4 protein expression in order to increase the cellular capacity for FA handling to accompany the increased cellular LCFA flux.

## Methods

### Subjects

Eight healthy males (age 30±1 yr; body weight 90.0±5.3 kg; body fat percentage 30.5±2.5; body mass index (BMI) 27.0±2.0) were recruited to the study (Table 1). These subjects were part of the individuals included in the study by Nybo *et al.* [40]. All subjects were non smokers and did not participate in regular physical activity (<1 time per week for the past 6 months, VO_2peak_ 3.4±0.1 l O_2_ min^−1^ or 38.2±1.8 ml O_2_ min^−1^ kg^−1^). Before volunteering for the study, all subjects were given full oral and written information about the course of the study and possible risks associated with participation. Written consent was obtained from each subject. The study was approved by The Copenhagen Ethics Committee (KF-01-203/03) and conformed to the code of ethics of the World medical Association (Declaration of Helsinki II).

**Table 1:** Subject characteristics.

	Pre Training		Post Training
Age, yr	30±1		30±1
Height, m	1.82±0.02		1.82±0.02
Body mass (BM), kg	90.0±5.3		87.5±5.8
BMI, kg m^−2^	27.0±2.0		26.8±2.2
Body fat, %	30.5±2.5	*	27.0±2.8
Lean body mass (LBM), kg	61.1±2.3	*	63.1±2.3
VO_2_ peak,			
l min^−1^	3.4±0.1	**	3.9±0.1
ml kg^−1^ BM min^−1^	38.2±1.8	**	45.7±2.0

Data are means ± SE.

*p<0.05;

**p<0.01 compared with Pre Training, *n = 8*.

Peak oxygen consumption (VO_2_ peak) was determined by an incremental exercise test until exhaustion on a Monark Ergomedic 839E bicycle ergometer (Monark, Varberg, Sweden). Expired air was collected in Douglas bags for later analysis of O_2_ and CO_2_ content. Subjects warmed up for 10 min at 100 Watt. Subsequently the resistance was increased by 25 Watt every min until the subject could not maintain the required intensity. The measured VO_2_ was accepted as maximal when no further increase in VO_2_ in response to an increased work load was obtained and when the respiratory exchange ratio (RER) was above 1.15. Body composition was measured using dual-energy X-ray absorptiometry (DEXA, LUNAR DPX IQ RBD, version 4.6b, WI, USA).

On a separate day subjects arrived at the laboratory after an overnight fast to perform an endurance test on bicycle ergometer. Exercise consisted of 120 min of bicycle exercise at 60% of each subject's individual VO_2_ peak. During the endurance test oxygen uptake and RER were frequently determined in order to evaluate the relative contribution of carbohydrate and lipid for energy turnover during exercise. Relative substrate utilization was calculated by following the equations; % Carbohydrate oxidation = ((RER-0.7)/0.3)×100. % Lipid oxidation = 100 - % Carbohydrate oxidation [41].

### Experimental day

After one day on a controlled standardized diet (65 Energy (E)% carbohydrate, 20 E% fat and 15 E% protein), subjects arrived in the morning in the laboratory after an overnight fast (12 h). Subjects rested for 30 min in the supine position after which blood was sampled from an arm vein. Thereafter a needle biopsy was obtained, using a Bergstrom needle with suction, from the vastus lateralis muscle under local anesthesia with 2% lidocaine. The muscle sample was quickly frozen (within 10 seconds) in liquid nitrogen and stored at −80°C until subsequent biochemical analyses.

### Training program

Then subjects initiated an 8 week supervised aerobic training program. The training was performed on a bicycle ergometer gradually increasing the training frequency during the 8 weeks. Each session lasted 40–90 minutes and consisted of intermittent exercise of varying intensities between 45–80% of VO_2_ peak. At three occasions during the training period, maximal oxygen uptake was measured on a bicycle ergometer and work load in the training sessions was adjusted accordingly.

At the end of the training period all initial tests were repeated. The endurance test was performed at the same absolute work load as pre-training (Table 2). Muscle biopsies were again obtained from the vastus lateralis muscle 48 h after the last exercise bout.

**Table 2:** Exercise performance parameters.

	Pre Training		Post Training
VO_2 (average)_			
l min^−1^	2.1±0.1	*	1.9±0.1
ml kg^−1^ BM min^−1^	24.2±1.7	*	22.4±1.2
HR _(average)_	151±6	**	131±6
RER _(average)_	0.90±0.03	*	0.85±0.01
% Lipid oxidation _(average)_	32.1±8.4	*	49.2±4.4
% CHO oxidation _(average)_	67.9±8.4	*	50.8±4.4

Data are means ± SE.

*p<0.05;

**p<0.01 compared with Pre Training, *n = 8*.

### Diet

During the training period instructions were given not to make any dietary changes. Initially as well as during the last week of the training period an individual detailed 3-day food registration was completed. The subjects weighed and recorded all food and beverages to the accuracy of 1 g. Subsequently energy intake and macronutrient composition of the diet were calculated (Dankost 2000, Danish Catering Center, Copenhagen, Denmark).

## Analyses

### Breath samples

Expired volumes of air was collected in Douglas bags and measured with a chain suspended Collins spirometer. A small sample of mixed expired air was analyzed for contents of O_2_ (VacuMed 17518A, Ventura, CA, USA) and CO_2_ (VacuMed 17515A, Ventura, CA, USA).

### Blood and plasma analysis

Blood glucose levels were measured using an ABL 615 analyser (Radiometer, Copenhagen, Denmark). Plasma long chain fatty acid (LCFA) concentration (NEFA C kit, Wako Chemicals GmbH, Neuss, Germany), plasma glycerol and plasma triacylglycerol (TG) (triacylglycerol GPO-PAP kit) were measured using enzymatic colorimetric methods (Hitachi 912 automatic analyzer, Mannheim, Germany). Plasma TG was calculated as plasma TG minus free plasma glycerol.

### Muscle biopsies

The muscle biopsies were immediately frozen in liquid nitrogen and stored at −80°C for subsequent biochemical analysis. Before biochemical analysis the muscle sample was freeze-dried and dissected free of all visible adipose tissue, connective tissue, and blood under a stereo microscope (Zeiss, Stemi 2000-C, Oberkochen, Germany). The dissected muscle fibers were pooled and then divided into sub-pools for the respective analyses.

### Muscle metabolites

Muscle glycogen concentration was determined as glycosyl units by a fluorometric method [42] after acid hydrolysis of freeze dried and dissected muscle tissue. IMTG content was determined by a biochemical method on dissected and freeze dried tissue as described previously [43].

### Activities of citrate synthase and 3-hydroxy-acyl-CoA dehydrogenase

Maximal activity of citrate synthase (CS) and 3-hydroxyacyl-CoA dehydrogenase (HAD) was measured at 25°C in freeze-dried muscle tissue in accordance to Lowry and Passonneau [44]. CS was measured on a spectrophotometer (Hitachi 912 Automatic Analyzer, Roche diagnostics, Hvidovre, Denmark) and HAD by fluorometry (Ratio-2 filter Fluorometer, Optical Technology Devices Inc., Bie & Berntsen, Roedovre, Denmark).

### Western blot

**Figure 1:**
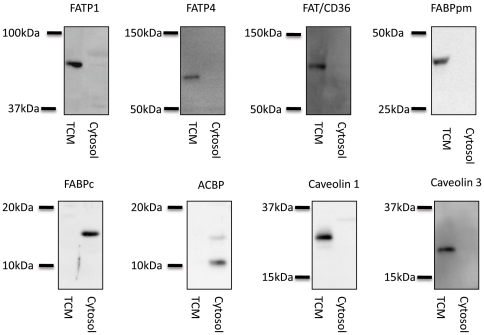
Representative images of the proteins analyzed in total crude membranes (TCM) and soluble cytocolic fractions (Cytosol) from human vastus lateralis muscle are shown. Protein concentration of TCM and cytosol fractions pre- and post training were determined and equal protein amounts were resolved by SDS-PAGE and membranes were immunoblotted using antibodies specific for FATP1; fatty acid transport protein 1 (63 kDa) and FATP4; fatty acid transport protein 4 (72 kDa), FAT/CD36; fatty acid translocase CD36 (88 kDa), FABPpm; membrane bound fatty acid binding protein (43 kDa), FABPc; cytoplasmic fatty acid binding protein (14 kDa), ACBP; acyl-CoA binding protein (10 kDa), and Caveolin 1 (22 kDa), Caveolin 3 (18 kDa). All Western blots were run in parallel with molecular weight markers. Relevant molecular weight markings above and below each analyzed protein are indicated.

Muscle proteins were analyzed in total crude membrane (TCM) and soluble protein (Cytosol) fractions by SDS-PAGE followed by immunoblotting. In short, muscle biopsies were homogenized at 0°C in a buffer (30 mM Hepes, 40 mM NaCl, 2 mM phenylmethylsulphonyl fluoride, 2 mM EGTA, 250 mM sucrose, 5 µM pepstatin A, 10 µg mL^−1^ aprotinin) for 20 seconds using a homogenizer (PT 3100; Brinkmann, Westbury, NY, USA). Homogenates were separated into a cytosolic (soluble protein fraction) and a membrane (TCM) fraction by centrifugation at 200.000 *g* for 1 h at 4°C. The pellet was resuspended (1 mM EDTA, 50 mM Tris Base, 4% SDS) and centrifuged at 4.000 *g* for 10 min. Protein content in the two fractions was measured by the bicinchoninic acid method (Pierce Chemical, Rockford, IL, USA) using bovine serum albumin as standards. Protein content was measured in triplicate and a maximal coefficient of variation of 5% between replicates was accepted. Equal amounts of protein from TCM and cytosolic fractions were loaded onto appropriate percentage polyacrylamide selfcasted gels (Bio-Rad, Herlev, Denmark) and transferred to polyvinylidene fluoride (PVDF)-membranes (IMMOBILION™ transfer membranes, Millipore Corporation, Bedford, MA, USA). The membrane was blocked for 1 hour at room temperature with skimmed milk or BSA in Tris-buffered saline containing 0.1% tween (TBST) and thereafter incubated overnight at 4°C with a primary antibody followed by incubation in horseradish peroxidase-conjugated secondary antibodies (DakoCytomation, Copenhagen, Denmark). After antigen-antibody complexes were visualized using enhanced chemiluminescence (IMMOBILION™ Western, Millipore Corporation, Bedford, MA, USA) and quantified using a Kodak Image station 2000MI (Kodak, Glostrup, Denmark) the signal was finally corrected for between-gel variation relative to a appropriate standard run on all gels. Preliminary experiments demonstrated that the amounts of protein loaded were within the dynamic range for the conditions used and the results obtained (data not shown). Representative Western blots of proteins in TCM and cytosol fractions analyzed in the present study are shown in figure 1.

### Antibodies

The following commercial antibodies were used: anti-CD36 (R&D Systems, Abingdon, UK), anti-Caveolin 3 (BD transduction laboratories, San Jose, CA, USA), anti-Caveolin 1 (BD transduction laboratories, San Jose, CA, USA), anti-β-actin (Sigma-Aldrich, Copenhagen, Denmark) and β-Tubulin (Sigma-Aldrich, Copenhagen, Denmark). The following non-commercial antibodies were used: anti-FATP4 (raised in rabbits [25]), anti-FATP1 (raised in rabbits [45]), anti-FABPpm and anti-FABPc (kindly donated by Prof. Glatz), and anti-ACBP (kindly donated by Dr. Faergeman).

### Statistics

Statistical evaluations were performed using SigmaPlot v.11.0 for windows. Data are presented as mean ± SE. To test for differences between Pre and Post training samples, the non parametric Wilcoxon's signed rank test for paired samples were performed. The strength of association between parameters was analyzed by Pearson product moment correlation analysis and 95% confident intervals (CI) for the obtained correlation coefficients were determined. Differences between samples and correlations between parameters were considered to be statistically significant when the p-value was less than 0.05.

## Results

### Diet

The average macronutrient composition of the diet was 53 E% carbohydrate, 30 E% fat and 17 E% protein and did not change during the experimental period (data not shown).

### Effects of training on whole body parameters

Body weight averaged 90.0±5.3 kg prior to exercise training and was unchanged after the training period (Table 1). In contrast lean body mass was significantly increased with exercise training from 61.1±2.3 kg to 63.1±2.3 kg (p<0.05; Table 1). The exercise training induced a 15% increase in peak oxygen uptake (VO_2_ peak) from 3.4±0.1 to 3.9±0.1 l min^−1^ (p<0.01; Table 1). Initially, resting blood glucose averaged 5.2±0.2 mmol l^−1^, plasma TG 1.8±0.4 mmol l^−1^ and plasma LCFA 378±58 µmol l^−1^, and remained unchanged after the training period.

### Lipid binding protein expressions in skeletal muscle

**Figure 2:**
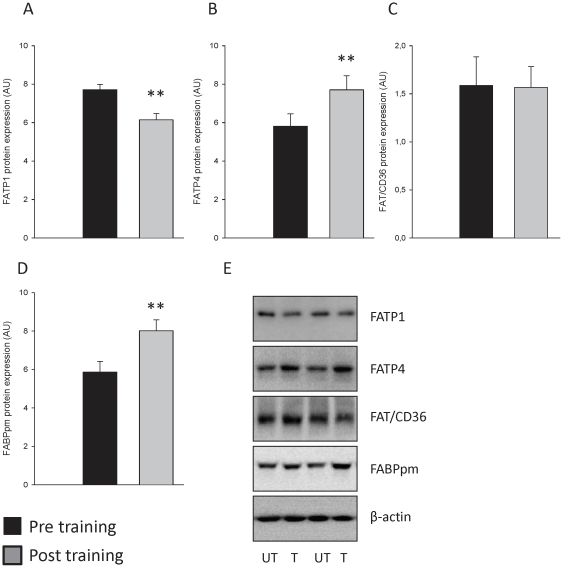
Divergent effects of exercise training on membrane bound lipid binding protein expression in human skeletal muscle. Protein concentration of total crude membrane (TCM) fractions before and after 8 weeks of exercise training were determined and equal protein amounts were resolved by SDS-PAGE and membranes were immunoblotted using antibodies specific for FATP1, FATP4, FAT/CD36 and FABPpm. β-actin was run on membranes as control and was unchanged with exercise training. Eight weeks of endurance exercise training resulted in reduced FATP1 protein expression in skeletal muscle (A). In contrast, (B) FATP4 and (C) FABPpm protein expression was increased after the training intervention compared to before training. FAT/CD36 protein expression was similar before and after training (D). Representative Western blots of protein levels before (Pre) and after the exercise training period (Post) from the same subjects are shown (E). Black bars represents pre training values and grey bars represents post training values. **; *p*<0.01.

**Figure 3:**
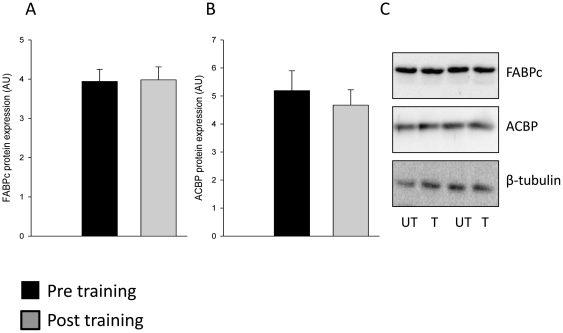
No response of exercise training on soluble lipid binding protein expression in human skeletal muscle. Eight weeks of endurance exercise training did not affect (A) FABPc or (B) ACBP protein levels in human skeletal muscle. Protein concentration of cytosol fractions pre- and post training were determined and equal protein amounts were resolved by SDS-PAGE and membranes were immunoblotted using antibodies specific for FABPc and ACBP. β-tubulin was run on membranes as control and was unchanged with exercise training. Representative Western blots of protein levels before (Pre) and after the exercise training period (Post) from the same subjects are shown (C). Black bars represents pre training values and grey bars represents post training values.

**Figure 4:**
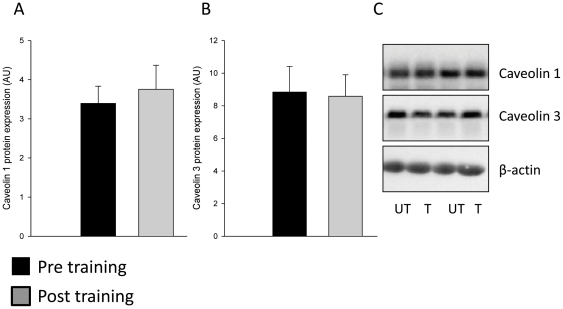
The Caveolae proteins Caveolin1 and Caveolin 3 were unaffected by exercise training. Eight weeks of endurance exercise training did not affect (A) Caveolin 1 and (B) Caveolin 3 protein levels. Protein concentration of TCM fractions pre- and post training were determined and equal protein amounts were resolved by SDS-PAGE and membranes were immunoblotted using antibodies specific for Caveolin 1 and Caveolin 3. β-actin was run on membranes as control and was unchanged with exercise training. Representative Western blots of protein levels before (Pre) and after the exercise training period (Post) from the same subjects are shown (C). Black bars represents pre training values and grey bars represents post training values.

Eight weeks of exercise training resulted in a 33% increase in FATP4 protein expression in skeletal muscle (p<0.01; Figure 2). In contrast, FATP1 protein expression was reduced by 20% (p<0.01; Figure 2). FABPpm protein expression was increased (37%, p<0.01), whereas FAT/CD36 protein expression remained unchanged after exercise training compared to the pre-exercise training period (Figure 3). The two cytosolic lipid binding proteins FABPc and ACBP remained unchanged after exercise training (Figure 3). In addition, exercise training did not affect protein levels of Caveolin 1 and Caveolin 3 (Figure 4), suggesting that the increased potential for LCFA utilization at rest and during submaximal exercise after exercise training seems not to depend on an increased skeletal muscle caveolae abundance.

*Muscle metabolites and mitochondrial enzymes*. Muscle glycogen content was increased with exercise training from 435±46 to 628±54 mmol kg^−1^ d.w. (p<0.05), whereas IMTG content (104±19 vs. 98±16 mmol kg^−1^ d.w.) on an average level, remained unchanged. Due to the lack of adequate muscle sample material, only 6 subjects were included in the IMTG analysis. The maximal activity of CS was increased by 36% (53.7±2.5 and 80.8±3.7 µmol g^−1^ min^−1^, before and after exercise training, respectively; p<0.01), and there was a tendency towards an increase in the maximal activity of HAD (25.8±1.5 and 30.9±3.7 µmol g^−1^ min^−1^; p = 0.078) after the training period.

### Correlations

**Figure 5:**
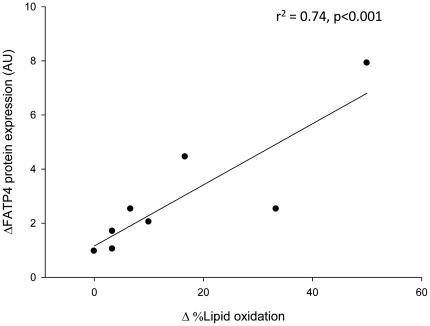
Association between lipid oxidation and exercise training induced adaptations in FATP4 protein expression in human skeletal muscle. Eight weeks of endurance exercise training resulted in a shift in substrate choice towards a higher FA utilization during submaximal exercise. This training induced adaptation was associated with the observed increase in FATP4 protein expression.

Since endurance exercise training resulted in a shift in substrate choice towards a higher FA utilization during submaximal exercise we tested whether the training induced adaptation in lipid binding protein expression was associated with changes in substrate utilization during exercise. The training induced adaptation in skeletal muscle FATP4 protein expression was associated with the increase in lipid oxidation during the endurance exercise test (r^2^ = 0.74; p<0.001; CI 0.408≤ρ≤0.972, Figure 5). No significant correlations between the changes in skeletal muscle FABPpm (r^2^ = 0.18; p = 0.3; CI 0.390≤ρ≤0.864) or FATP1 (r^2^ = 0.40; p = 0.1; CI -0.118≤ρ≤0.922) protein expression and lipid oxidation during the endurance test were observed.

## Discussion

In the present study we demonstrated that FATP1 and FATP4 are expressed in human skeletal muscle on a protein level. Furthermore, we showed that FATP4 protein content was increased by 33% in skeletal muscle after 8 weeks of endurance exercise training compared to pre-training levels. In contrast, FATP1 protein content was reduced by 20%. Interestingly, the observed increase in FATP4 protein content was associated with an increased lipid oxidation during prolonged submaximal exercise.

Functional studies using primary adipocytes and skeletal muscle from FATP1 null mice [23] or FATP1 knockdown 3T3-L1 adipocytes [46] have shown that FATP1 only plays a minor role in basal FA uptake/transport. In contrast, FATP1 was shown to be required for insulin stimulated LCFA uptake in adipocytes [46], [47], which coincided with an insulin stimulated translocation of FATP1 to the plasma membrane [47]. In addition, FATP1 KO mice showed a robust reduction in high fat diet induced accumulation of TG and diacylglycerol (DG) in skeletal muscle [48]. Deletion of one allele of FATP4 resulted in a 48% reduction in FATP4 protein level and a corresponding 40% reduction in fluorescently labeled LCFA (BODIPY) uptake in isolated enterocytes [2]. Milger and coworkers observed a net increase in LCFA influx when overexpressing FATP4 in COS cells [25], whereas FATP4 knockdown in adipocytes showed no effect on neither basal nor insulin stimulated FA uptake [49]. In the latter study it was suggested that FATP4 may have a functional role in FA re-esterification following lipolysis [49]. Moreover, in contrast to FATP1, both Milger et al. *(2006)* and Lobo et al. *(2007)* showed that FATP4 was not localized to the plasma membrane [25], [49], but rather to the endoplasmatic membranes [25], suggesting that FATP4 may be a key intracellular regulator of LCFA handling. Adding to the complexity, recent studies have suggested, that in addition to the importance in LCFA uptake and esterification, FATP1 may play a novel role in mitochondria [50], [51], [52]. To this end, new and interesting findings by Hagberg and coworkers point towards a key role of the surrounding vasculature in the regulation of LCFA uptake into peripheral tissue in a FATP3 and FATP4 dependent manner [53]. Thus, regulation of LCFA uptake into peripheral tissue, such as skeletal muscle, seems to be governed at multiple sites.

In the present study both FATP1 and FATP4 protein content in human skeletal muscle were affected by 8 weeks of exercise training in association with an increase in skeletal muscle oxidative potential, supported by the finding of an increased activity in mitochondrial key enzymes. Surprisingly, FATP1 and FATP4 protein expression were found to be oppositely regulated in skeletal muscle (Figure 2). This could suggest that these two proteins have different roles in the cellular handling of LCFA in human skeletal muscle, which is in line with findings in some cell models [49]. Interestingly, we observed an association between the increase in FATP4, but not FATP1, protein expression in skeletal muscle and the oxidation of LCFA (estimated from RER) during a submaximal exercise bout (Figure 5). This suggests a role of FATP4 in skeletal muscle LCFA oxidation during exercise. Importantly, these associations are based on a low number of subjects and should thus be interpreted with caution. Findings from FATP1 KO mice have shown a robust reduction in TG and DG accumulation in skeletal muscle when consuming a high fat diet [48], and thus the role of FATP1 could be to handle LCFA taken up into the myocyte. In contrast to FATP1 protein it seems that FATP4 protein plays a role in intracellular LCFA trafficking. This suggestion is supported by the findings from the present study of a positive association between FATP4 protein level and lipid oxidation during exercise and by the findings by Lobo and coworkers [49], who showed that FATP4 may function in LCFA re-esterification following lipolysis in adipocytes. Therefore, one could speculate that in human skeletal muscle FATP4 is important in handling LCFA released from lipid droplets by directing them either to re-esterification into the lipid droplets or oxidation in mitochondria, whereas, the role of FATP1 could be handling LCFA taken up into the myocyte. Furthermore, earlier studies on FATP proteins have demonstrated, that these proteins shared considerable sequence homologies and domain organization with acyl-CoA-synthethase, suggesting that FATP proteins are members of the super-family of adenylate forming acyl-CoA synthethases (ACS), which are responsible for the formation of the fatty acyl-CoA esters from LCFA that are needed for LCFA to be metabolized [4], [8], [54], [55]. Taken together, the observations made in the present study supports differential functions for FATP1 and FATP4 proteins in regulation of LCFA handling in human skeletal muscle, likely by acylation of incoming LCFA and LCFA generated from IMTG lipolysis, and directing these to metabolism or esterification. The distribution of FATP1 and FATP4 between different muscle fibers is unknown in human skeletal muscle. It is unlikely that the applied training regime will induced a shift from type 2 fibers to type 1 fibers in humans [56], and thus we do not believe this is a contributing factor in the differential adaptation in FATP1 and FATP4 protein expression. However, it should be noted that a training induced shift within type 2 fibers (i.e. from type 2× to type 2A) could have occurred. If this in some way affects FATP1 and/or FATP4 protein expression warrants further study. In the present design the subjects were examined before and after 8 weeks of exercise training. Due to the lack of a non exercising control group we cannot rule out that external factors other than exercise training and diet could have influenced our findings. Therefore, the present findings should be corroborated in a longitudinal study including a non-exercising control group. On the other hand, different adaptations of several of the measured proteins to the 8 week intervention period were observed supporting that the present findings occurred as a consequence of exercise training.

In the present study endurance exercise training induced an increase in FABPpm protein content. This is consistent with several findings in the literature of increased FABPpm protein expression in vastus lateralis muscle with different types of exercise training [16], [19], [20], [21]. In contrast, findings from studies where the effect of exercise training on skeletal muscle FAT/CD36 protein expression have been investigated in humans are contradictory [19], [20], [21], [30]. In the present study FAT/CD36 protein levels in the vastus lateralis muscle remained unchanged after 8 weeks of endurance training compared to pre training levels in male human subjects (Figure 2). These findings are in agreement with some previous studies [19], [20], but not all [21], [30]. In a cross sectional study, comparing trained and untrained male and female subjects, Kiens *et al. (2004)* similar protein levels of FAT/CD36 in vastus lateralis muscle were observed in the trained and untrained groups, despite the markedly higher oxidative capacity in the trained subjects [19]. In contrast, Tunstall *et al. (2002)* reported that 9 days of endurance exercise training induced an increase in FAT/CD36 protein expression by ∼20% in skeletal muscle of 3 men and 4 women [30]. Importantly, 90 min of submaximal bicycle exercise (60% of VO_2_ peak) induced an immediate increase in FAT/CD36 mRNA in vastus lateralis muscle from both untrained and trained males and females [19]. Furthermore, 24 hours after a single bout of glycogen depleting exercise FAT/CD36 protein expression was significantly elevated in male subjects [57]. Therefore, the increase in FAT/CD36 protein expression by Tunstall *et al. (2002)* could be ascribed to the effect of the last exercise bout rather than a training effect, because the muscle biopsy in that study was obtained only 24 hours after the last exercise bout [30]. Spriet and co-workers recently observed that FAT/CD36 protein content was either unchanged or increased by ∼10% in vastus lateralis muscle after 2 and 6 weeks of high intensity interval bicycle exercise training (10 bouts of 4 min at >90% VO_2_max followed by 2 min rest) [20], [21]. The reason for the discrepancy in findings by the latter studies applying similar protocols is unclear. It is however interesting to note that FAT/CD36 protein content have only been shown to increase when short term high intensity exercise training was performed [21], [30] and not when more aerobically endurance exercise training, as in the present study, was applied [19]. The up-regulation of FAT/CD36 protein seen in some human studies [21], [30] and also in some rat studies, using 7 days of chronic low frequency stimulation of the peroneal nerve [58], [59], could be an early adaptation in FAT/CD36 protein levels, whereas long-term endurance exercise training gradually would return the FAT/CD36 protein content to the initial levels, possible after other adaptations in skeletal muscle metabolism have occurred. The observed differences could also potentially be the result of the different type of exercise (endurance vs. high intense exercise) or subject recruitment criteria, such as pre-training oxygen uptake and body composition.

In summary, in the present study we showed an up-regulation of FATP4 protein expression in human skeletal muscle after 8 weeks of endurance training. The increase in FATP4 protein was associated with an increased lipid oxidation during prolonged submaximal exercise after the training intervention. This possible interaction between FATP4 and lipid oxidation should be corroborated in a study using a larger cohort of subjects including a non-exercise control group. In contrast, the endurance exercise training intervention was followed by a decrease in FATP1 protein expression. Taken together, it is demonstrated that both FATP1 and FATP4 proteins are present in skeletal muscle and the data suggest that FATP1 and FATP4 proteins could have different roles in handling fatty acids within human skeletal muscle.
